# Aerosolized Hypertonic Saline Hinders Biofilm Formation to Enhance Antibiotic Susceptibility of Multidrug-Resistant *Acinetobacter baumannii*

**DOI:** 10.3390/antibiotics10091115

**Published:** 2021-09-15

**Authors:** Hui-Ling Lin, Chen-En Chiang, Mei-Chun Lin, Mei-Lan Kau, Yun-Tzu Lin, Chi-Shuo Chen

**Affiliations:** 1 Department of Biomedical Engineering and Environmental Sciences, National Tsing Hua University, Hsinchu 300044, Taiwan; huiling@cgu.edu.tw (H.-L.L.); wendy19951208@gmail.com (Y.-T.L.); 2 Department of Respiratory Therapy, Chang Gung University, Taoyuan 33323, Taiwan; paulz60610@gmail.com; 3 Department of Respiratory Care, Chang Gung University of Science and Technology, Chiayi 61363, Taiwan; 4 Department of Respiratory Therapy, Linkou Chang Gung Memorial Hospital, Taoyuan 33305, Taiwan; lmc0819@gmail.com (M.-C.L.); himeilan@gmail.com (M.-L.K.)

**Keywords:** multidrug-resistant *Acinetobacter baumannii* (MDR-AB), hypertonic saline, antibiotics, biofilm, aerosol delivery

## Abstract

Limited therapeutic options are available for multidrug-resistant *Acinetobacter baumannii* (MDR-AB), and the development of effective treatments is urgently needed. The efficacy of four aerosolized antibiotics (gentamicin, amikacin, imipenem, and meropenem) on three different MDR-AB strains was evaluated using hypertonic saline (HS, 7 g/100 mL) as the aerosol carrier. HS aerosol effectively hindered biofilm formation by specific MDR-AB strains. It could also interrupt the swarming dynamics of MDR-AB and the production of extracellular polymeric substances, which are essential for biofilm progression. Biofilms protect the microorganisms from antibiotics. The use of HS aerosol as a carrier resulted in a decreased tolerance to gentamicin and amikacin in the biofilm-rich MDR-AB. Moreover, we tested the aerosol characteristics of antibiotics mixed with HS and saline, and results showed that HS enhanced the inhaled delivery dose with a smaller particle size distribution of the four antibiotics. Our findings demonstrate the potential of using “old” antibiotics with our “new” aerosol carrier, and potentiate an alternative therapeutic strategy to eliminate MDR-AB infections from a biofilm-disruption perspective.

## 1. Introduction

Hospital-acquired pneumonia is most commonly caused by multidrug resistant Gram-negative bacteria, such as *Acinetobacter baumannii*, *P. aeruginosa*, and *K. pneumonia*. Among these, *A. baumannii* is one of the most common causative pathogens [1]. *A. baumannii*, a Gram-negative coccobacillus, is a leading cause of severe nosocomial infections in the current health system [2]. *A. baumannii* is the primary agent associated with pneumonia, septicemia, endocarditis, meningitis, and urinary tract infections [3]. *A. baumannii* can be intrinsically resistant to many commonly used antibiotics, such as aminopenicillins, first-generation and second-generation cephalosporins. The increasing prevalence of multidrug-resistant *A. baumannii* (MDR-AB) infection has emerged worldwide due to prolonged hospital stays [4,5]. A few mechanisms are suspected in the development of MDR-AB, including altered membrane permeability, mutations in efflux pumps and aminoglycoside-modifying enzymes, and the expression of β-lactamases [3]. With the high prevalence of MDR-AB, combination therapy is frequently used to decrease the risk of resistance and improve patient outcomes [6].

Inhaled antibiotics allow rapid and direct delivery to the lungs at a high concentration [7]. To treat bacterial pneumonia effectively, antibiotics must reach the minimum inhibitory concentration in the region of the infected site. However, with drugs administered systemically, the drug concentration in the lungs depends on the drug’s ability to penetrate the alveolar capillaries. For example, fluoroquinolones can highly penetrate the lungs compared to other β-lactams or colistin, as colistin has low penetrance through the capillaries [8,9,10]. Routinely inhaled antibiotics have been the standard treatment for patients diagnosed with cystic fibrosis infected with *P. aeruginosa* [11]. Previous studies have illustrated that adjunct aerosolized colistin to treat patients with MDR-AB had better eradication and cure rates than intravenous administration alone [12,13].

Biofilm formation may alter the responses of the microbial community to antibiotic agents, and contribute to drug resistance challenges [14,15,16]. The formation of biofilm is composed of several steps: First, through the secreted extracellular polymeric substances (EPSs) on the cell surface, bacteria attach to the solid surface. Under proper conditions, the attached bacteria cluster together as a complex multicellular community with an EPS matrix. In addition to providing anchorage for bacteria, the biofilm matrix provides a microenvironment with a unique nutrient gradient and oxygen conditions, which may stimulate various physiological functions of the bacteria, such as dormancy and intercellular communication in biofilms [16]. Although various mechanisms have been proposed to interpret the altered properties of bacteria in biofilms, it is widely recognized that the biofilm matrix can enhance the survival of bacteria to antimicrobials.

MDR of *A. baumannii* is highly associated with biofilm formation [14]. Studies have shown that biofilms prompt adherence to the host and abiotic surfaces and enhance the survival rate of bacteria to antibiotics. For *A. baumannii*, biofilms can shield bacteria from therapeutic agents and stimulate intercellular communication for colonization [17]. Since the efficacy of antimicrobial agents against microbes is highly dependent on the integrity of the biofilm matrix [15,16], interfering with the biofilm structure is a promising approach to eliminate the protection of the matrix from antibiotics. Different pharmacological approaches and engineering methods have been proposed to disrupt biofilms and eliminate microbial MDR [18]. However, due to the complicated composition of biofilms, such as EPS polymers, proteins, and DNA fragments [15,19], an effective approach for biofilm destruction remains challenging in the biomedical field.

This study aimed to eliminate biofilm formation of *A. baumannii* to recover the therapeutic efficacy of antibiotic agents. We speculated that the delivery of aerosolized hypertonic saline (950 mM sodium chloride) can modulate the ionic concentration of the bacterial microenvironment, and we explored the potency of hypertonic saline for MDR-AB therapy. By replacing the divalent ions within the biofilm matrix [15], high concentrations of mono-ions are expected to interrupt the biofilm of MDR-AB on abiotic surfaces. Four antibiotics that are commonly used in combinational therapies for *A. baumannii* infections were selected: imipenem, meropenem, amikacin, and gentamicin [6]. In addition to the direct impact on the biofilm structure and microbial colonization, we further evaluated the influence of aerosol hypertonic saline on the extracellular secretion of MDR-AB during the colonization process. By regulating the microenvironment of MDR-AB using aerosol hypertonic saline, our approach should provide an alternative perspective for current therapeutic agents to overcome MDR *A. baumannii* and its related diseases. 

## 2. Materials and Methods

### 2.1. Bacteria Culture and Drug Resistance Test

Three MDR-AB strains were used in this study. MDR-AB isolates (14B0087, 14B0091, and 14B0094) were purchased from the Bioresource Collection and Research Center, Taiwan, following the biosafety protocol.

Bacterial colonies were routinely subcultured with Difco™ Tryptic Soy agar (TSA) plates. Cells were inoculated in 3 mL liquid culture, using Difco™ Tryptic Soy Broth (TSB), and grown at 37 °C in a shaking incubator overnight (14–16 h). The overnight liquid cultures with optical density (OD_600_) exceeding 1.0 were used for subsequent experiments.

**Figure 1:**
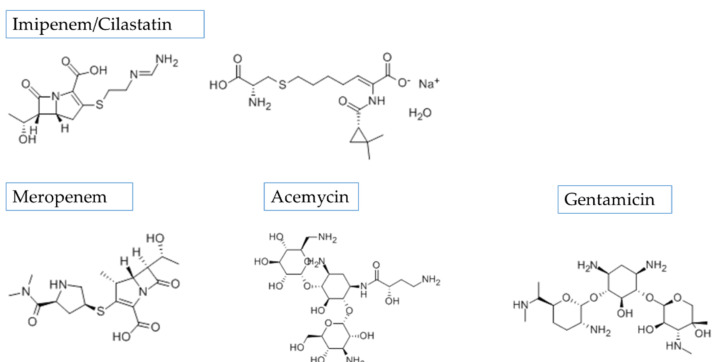
Chemical structure of four antibiotics used in this study.

Four antibiotics agents were used to evaluate the drug response of MDR-AB: Imipenem/Cilastatin (500 mg/500 mg, Facta Farmaceutici, Italy), Meropenem (250 mg, Sumitomo Dainippon Pharma Co., Ltd., Japan), acemycin (Amikacin 125 mg/mL, Yung Shin Pharmaceutical Ind. Co. LTD, Taichung, Taiwan), and gentamicin (40 mg/mL, Tai Yu Chemical & Pharmaceutical Co., LTD, Hsinhsu, Taiwan)]. The chemical structures of the four antibiotics listed are presented in Figure 1. Antibiotic agents were aerosolized with either regular saline (135 mM NaCl) or hypertonic saline (950 mM). The resistance tests were revised according to a previous study. In brief, 2 µL of overnight liquid culture (OD_600_ = 1.0) was dripped onto TSA. Thereafter, 4 mL of antibiotic at the desired concentration was aerosolized and deposited onto the specimens. Approximately 0.2 µL of antibiotic solution was deposited on the bacteria culture. After aerosol treatment, the agar plate was incubated at 37 °C overnight (14–16 h). MDR-AB grew into visible colonies after overnight incubation. Selected colonies were scooped into 1 mL of 1× phosphate-buffered saline (PBS); the colony was dispersed and further diluted to 1 × 10^5^–10^9^ in 1× PBS. The diluted cells (20 µL) were spread onto TSA agar plates and incubated overnight for colony formation unit quantification. The survival rate of MDR-AB was determined by quantifying the number of recovered colonies on the plates, and normalized with aerosolized saline treatment for each independent experiment.

### 2.2. Aerosol Generation and Aerosol Particle Size Distribution

A 2.5 L closed aerosol delivery chamber was designed, and the culture dishes were placed in the middle of the chamber. Antibiotic selection by the Bioresource Collection and Research Center, Taiwan, was based on sensitivity and susceptibility, and the dose was determined as the maximum inhibition of bacterial motility. Imipenem (7.81 mg), meropenem (31.25 mg), acemycin (7.81 mg), and gentamicin (10 mg) were used. A pneumatic jet nebulizer (Besmed Inc., Taipei, Taiwan) powered by a 50 psi compressed oxygen flow at 8 L/min was filled with 4 mL antibiotics mixed with 0.9% saline or 7% hypertonic saline. Nebulization was stopped by sputtering, and the culture dish was removed for testing 30 s later (*n* = 5).

Aerosol characteristics were determined by a cascade impaction, according to the United States and European Pharmacopeia recommendations. A Next-Generation Impactor (Copley Scientific Limited, Nottingham, United Kingdom) was assembled with internal and external filters and placed in a temperature-controlled chamber at 4 °C. The impactor was calibrated at 15 L/min using a mass flow meter (TSI Cooperation, Shoreview, Minnesota). The mass median aerodynamic diameter (MMAD), geometric standard deviation (GSD), and fine-particle fractions (percentage of particles < 5 mm) were calculated using the CITDAS 3.1 software (Copley Scientific, Nottingham, United Kingdom). The dry weight of the nebulizers was taken after loading 4 mL of the selected antibiotic mixture, and after the completion of nebulization. The emitted dose of the nebulizer was calculated as the difference between the loaded and post-nebulization weights.

### 2.3. Biofilm Quantitative Measurement and Scanning Electron Microscopy

To evaluate the influence of the hypertonic saline on biofilm formation, overnight cultures were diluted to OD_600_ = 0.01, with the TSB containing either 135 nM NaCl or 950 mM and deposited in 96-well plates at 200 μL/well. After incubation at 37 °C for 48 h, the biofilm was gently washed with 1× PBS to remove excessive suspended cells. Thereafter, the specimens were stained with 0.5% crystal violet (*w*/*v*) for 10 min. The stained biofilm was dissolved in 95% ethanol, and biofilm formation was quantified by measuring OD_550_ with a spectrometer.

A 12 × 12 mm glass circle coverslip was set at an angle of 30° to 50° in a flat-bottom 24-well plate. Diluted overnight liquid cultures at 1:100 in TSB were carefully added at 300 μL/well. The cells were incubated for 48 h. After 48 h, the circle coverslips were carefully moved from the well, and the cells were prepared for visualization using scanning electron microscope (SEM). Biofilms were first fixed in 4% glutaraldehyde for 20 min, and then serially diluted with ethanol. Critical point drying was performed before coating 2 nm of gold onto the specimen surface. The prepared specimens were imaged using SEM.

### 2.4. The Spatial Distribution of Protein and Carbohydrate in Biofilm

MDR-AB colonies were prepared as described above. The prepared colonies were treated with experimental aerosols and incubated at 37 °C for 4 h. To identify the protein/carbohydrate distribution, WGA (Wheat Germ Agglutinin, 1 mg/mL) stained carbohydrates, and SYPRO™ Ruby Biofilm Matrix Stain (used as manufacturer’s suggestion), stained proteins in the biofilm, were applied. Stained biofilms were observed using laser scanning confocal microscopy at 20× magnification.

### 2.5. The Dynamic of Bacteria Swarming

Bacterial swarming assays were performed on a substrate containing 0.3% agarose (semisolid surface) in 3 µL of TSB overnight culture (OD_600_ = 0.3). MDR-AB was dripped onto the surface of 0.3% agar. Bacterial swarming was monitored for 10 h at 37 °C using time-lapse microscopy (interval = 1 image/30 min).

### 2.6. Statistical Analysis

All data are presented as the mean± standard deviation (SD). Comparisons among groups were conducted using one-way analysis of variance (ANOVA) with post hoc Bonferroni correction. A Student’s two-tailed *t-*test was used to determine the significance of the difference between the mixture of 7% hypertonic saline or 0.9% saline, and the results are indicated as * *p* < 0.05, ** *p* < 0.01, and *** *p* < 0.001.

## 3. Results and Discussion

### 3.1. Hypertonic Saline Hinders the Biofilm Formation of A. baumannii

**Figure 2:**
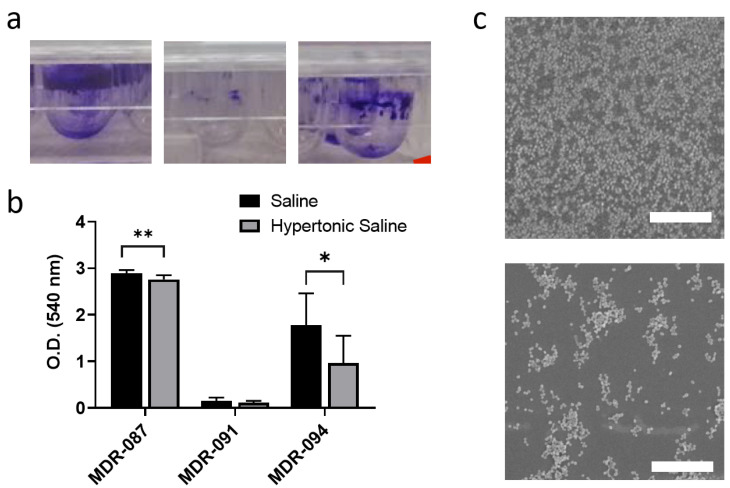
The biofilm of *A. baumannii*: (**a**) images of crystal violet staining to show the biofilm formed by MDR-AB; (**b**) quantitative measurement of biofilm formation using a spectrometer; (**c**) SEM images of colonized MDR-087 at the air–liquid interface with aerosolized saline (top) and aerosolized hypertonic saline (bottom). ** *p* < 0.05; * *p* < 0.001.

Biofilms provide protection from antibiotic agents to the microbial community. The stickiness of EPS plays an essential role in the structure formation of the microbial community. A previous study showed that the stickiness of EPS of Sagutula can be altered by ionic strength in the surrounding microenvironment [20]. As a critical substance for bacteria to adhere to the surface, the influence of ionic strength on biofilm formation by *A. baumannii* has not yet been fully explored. We first tested whether the treatment of hypertonic saline aerosol could eliminate biofilm formation on the solid surface with three MDR *A. baumannii* strains. First, we cultured *A. baumannii* in a medium containing 950 mM sodium chloride to recapture high ionic strength in the microenvironment. After 48 h of culture, the biofilm was labeled with crystal violet staining, and different biofilm patterns were observed over these three MDR strains (Figure 2a). An abundant biofilm was formed with MDR-087, and there was almost no biofilm formation with MDR-091. After culturing in a medium containing 950 mM NaCl, MDR-094 showed approximately 54% reduced biofilm formation, and the biofilm of MDR-087 also showed a slight decrease (Figure 2b). The results indicated that a high concentration of NaCl could help to limit *A. baumannii* biofilm formation.

Furthermore, SEM was used to examine the microstructures of the biofilm. From the images, we observed a more fractural biofilm structure after treatment with the hypertonic aerosol. *A. baumannii* was separated into small clusters, rather than colonizing together (Figure 2c). Combining the alteration of the biofilm microstructure and quantitative biofilm assay at the macroscale, our data demonstrated that hypertonic saline aerosol hinders biofilm formation and disperses microbial colonization. The biofilm matrix is an important abiotic factor that influences the antibiotic resistance of microbes [14,16]. In comparison to dense biofilms, antibiotic agents can easily penetrate the fractal matrix and kill bacteria within it. Although the dosage of hypertonic saline is not enough to eliminate bacterial growth through osmotic stress, our data implied that the abolishment of dense biofilm formation may enhance the efficacy of antibiotic agents.

### 3.2. Hypertonic Saline Stimulates Bacterial Swarming and Alters the Distribution of EPS

**Figure 3:**
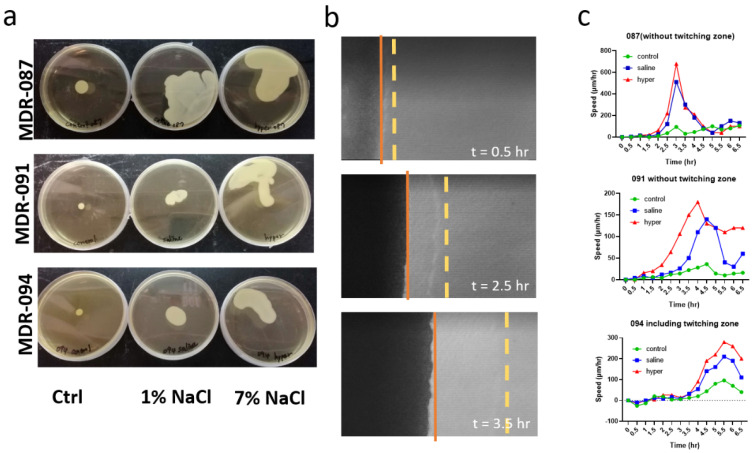
The swarming dynamics of MDR-AB: (**a**) pictures of MDR-AB strains swarming on soft agar plated after 72 h incubation; (**b**) time-lapse microscopy images of active twitching movement of MDR-AB; (yellow dashed line: front edge of the twitching layer; orange line: back edge of the twitching layer); (**c**) quantitative analysis of MDR-AB swarming under various experimental conditions.

We examined the potential mechanisms underlying the decrease in biofilm formation. A previous study illustrated a negative correlation between bacterial motility and biofilm formation [20]; as the first step of biofilm formation, microbes slow down and adhere to the substrate. Thus, we speculated that hypertonic saline can stimulate the movement of bacteria and decrease the adhesion of bacteria to the substrate. The inhibition of motility promotes biofilm formation. The observed increasing motility of *A. baumannii* implies an unstable adherence between bacteria and substrate, which can contribute to the loss of biofilm formation [21]. The results of the swarming assay showed that the swarming motility of all three MDR strains significantly increased after the aerosol treatments, either with saline or with hypertonic saline (Figure 3a). To further determine the difference between aerosolized saline and aerosolized hypertonic saline treatment, we measured the swarming dynamics of *A. baumannii* using time-lapse microscopy. Under microscopy, active bacterial twitching in the transparent leading-edge surrounding the colony was observed (Figure 3b).

Following the rapid expansion of the lead edge, a three-dimensional colonized bacterial community formed gradually, which was shown to expand the opaque portion under phase microscopy (Figure 3b). In the presence of hypertonic saline aerosol, we observed an increasing swarming movement of *A. baumannii* (Figure 3c). For instance, for the MDR-091 group at t = 3.5 h after seeding, the data showed that the velocity of swarming velocity increased from 22 to 50 µm/h with saline treatment and further increased to 150 µm/h with hypertonic saline treatment.

The EPSs, serving as the fundamental material of biofilm, critically influence the adhesion of bacteria to the surroundings [22]. Previous studies have shown that increasing the mono-ionic concentration enhances the stickiness of EPSs extracted from *E. Huxieyi* and *Amphora* sp. [20]. Our data on unstable adhesion and high motility suggested that the high concentration of monotonic ions may cover the negative surface charge of EPS and reduce the entanglement of *A. baumannii* EPSs. On the other hand, a further increase in salt concentration can lead to decreased motility. Studies have also shown the limited motility of *A. baumannii*, due to the increased surface hydrophobicity under high salt conditions (300 mM in 0.5% agar); excessive hypertonic saline aerosol may also lead to undesired physiological responses of patients, such as coughing. More detailed experiments are required for dose optimization before clinical respiratory therapy.

The hypertonic saline aerosol may directly reduce the entanglement of the EPS matrix and change the EPS secretion of bacteria [23]. It is known that microorganisms produce different EPSs under different environmental stresses. In contrast to the salt concentration (~400 mM) reported in other osmotic stress studies, our experimental deposited NaCl dosage (approximately 150 mM) may not lead to significant osmotic stress in *A. baumannii*. However, aerosolized hypertonic saline can locally alter the ionic concentration at the deposition sites [24] and potentially regulate the EPS secretion of *A. baumannii*.

**Figure 4:**
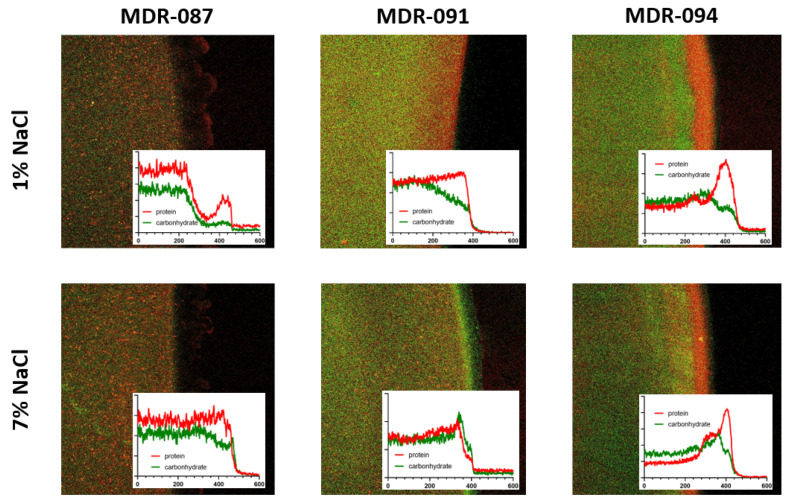
The spatial distribution of protein/carbohydrate composition in biofilm matrix. Represented confocal images of colony edge under different treatments. Proteins (red) and carbohydrate were labeled with fluorescein. Insets: Intensity profiles of protein staining (red) and carbohydrate labeling (green) cross the colony edge.

By applying laser scanning confocal microscopy with protein/carbohydrate labeling assays [15,25], we reconstructed the spatial distribution of protein/carbohydrate in the fast-progressing colony edges to evaluate the influence of aerosol treatment on EPS production. The results showed different compositions at the colony edge of the three MDR strains (Figure 4). MDR-087 showed the lowest protein content compared to the other two MDR strains. A remarkable protein/carbohydrate segregated distribution was observed in MDR-091 and MDR-094, and a 50 µm-thick distinguished carbohydrate layer was found at the edge of the MDR-094 colony. When treated with hypertonic saline aerosol, all three strains seemed to express more carbohydrate-rich EPS (as shown in the insets). A more segregated protein/carbohydrate distribution was observed in MDR-091 and MDR-094, and a thin layer of proteins was present in the EPS layer at the edge of MRD-091 colony.

Our data showed that the deposited hypertonic saline aerosol could regulate the EPSs produced by *A. baumannii.* Extracellular proteins and carbohydrates are known to play important roles in the maintenance of biofilm matrices. It has also been shown that a lack of protein is expressed on the cell surface, and *A. baumannii* cannot colonize the epithelium of the respiratory airway. In this study, high carbohydrate expression implied a lower colonizing capacity of *A. baumannii* after aerosolized hypertonic saline treatment. Although we evaluated the composition using bacterial colonies on soft agar surfaces, the lower protein/carbohydrate ratio may also support the formation of fractured colonies at the liquid–air interface (Figure 2c). Moreover, the hydrophobic interactions between proteins can contribute to the stability of the biofilm matrix, and the sticky EPSs are associated with a higher protein to carbohydrate ratio of EPSs [20,25]. The observed alteration of protein/carbohydrate in the biofilm matrix implied lower adhesion for the bacteria within the matrix, showing higher motility.

**Figure 5:**
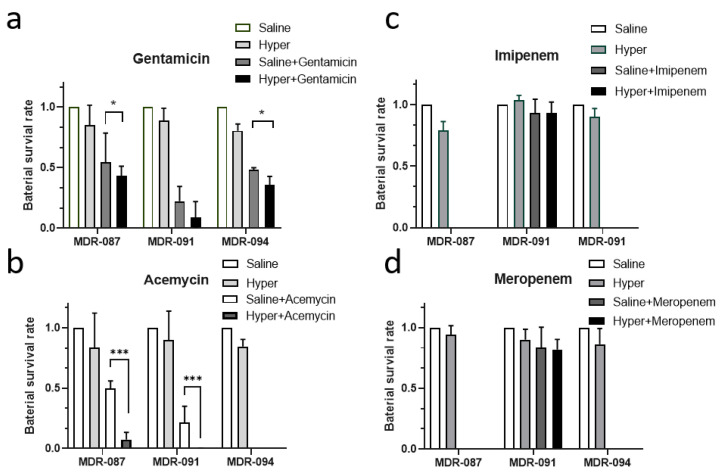
The antibiotic response of MDR-AB. (**a**–**d**) Different antibiotics were aerosolized with either saline or hypertonic saline and were deposited on MDR-AB strains. * *p* < 0.05; *** *p* < 0.001.

Since biofilm formation can contribute to the antibiotic resistance of microorganisms, after identifying the impact of hypertonic saline aerosol on biofilm formation, we aimed to study the influences of aerosol hypertonic saline on the antibiotic resistance of MDR-ABs. First, we observed the enhancing efficacy of gentamicin and acemycin on MDR-087, which is known to show intermediate resistance to gentamicin (Figure 5a,b). For MDR-094, which is resistant to gentamicin, a similar effect of hypertonic saline aerosol for decreasing antibiotic resistance was observed (Figure 5a). However, for MDR-091, which is resistant to imipenem and meropenem, aerosolized hypertonic saline showed no significant influence on suppressing the bacterial survival rate under antibiotic treatments (Figure 5c,d). Together with the biofilm quantification measurement, we observed that MDR-091 produced the lowest biofilm under both experimental conditions compared to MDR-087 and MDR-094. Thus, biofilm formation does not seem to contribute to the drug resistance of MDR- 091, and the interruption of biofilm formation showed no impact on MDR-091.

### 3.3. Influence of Hypertonic Saline to Aerosol Characteristics and Delivered Dose

**Figure 6:**
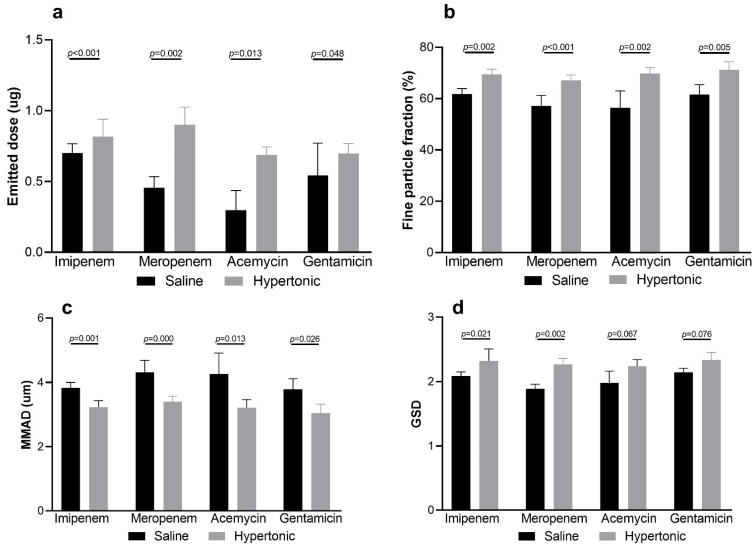
Comparisons of aerosol characteristics: (**a**) emitted doses were higher using hypertonic saline mixtures of all four antibiotics; (**b**,**c**) hypertonic saline generates significantly higher fine particle fraction; (**b**) resulting smaller MMAD; (**d**) the geometric standard deviation of imipenem and meropenem mixed with hypertonic saline was significantly greater.

Changing the diluent in aerosol treatment may change the characteristics of aerosols and lead to alterations in the inhaled dosage [26]. An impactor was used to simulate physiological inhalation/exhalation to determine the aerosol characteristics. Our study showed that hypertonic saline as a diluent for antibiotics altered the emitted dose and particle size distribution (Figure 5). Compared to normal saline, the emitted doses with hypertonic saline–antibiotic mixtures were significantly greater (Figure 6a), indicating that hypertonic saline might carry higher antibiotic doses to the lungs. Furthermore, the smaller MMAD and larger fine particle fraction would facilitate antibiotics in the peripheral regions of the lungs where pneumonia is most likely to occur (Figure 6b,c). Previous studies have proven the safety of hypertonic saline, and nebulized hypertonic saline alone for patients with airway hypersecretions, such as cystic fibrosis and COPD [27]. Additionally, hypertonic saline reduces inflammation in the respiratory systems [28]. Our study demonstrated that using hypertonic saline as a diluent of antibiotics before nebulization facilitates greater drug delivery to the peripheral regions, and further studies of drug deposition confirmed by radiolabeled or clinical outcomes are warranted. Moreover, considering the interactions between drug molecules and solvent, more detail studies are required to determine pharmacological characteristics, such as degradation and hydrolysis, while applying hypertonic saline with specific drug molecules.

## 4. Conclusions

This study demonstrated that aerosolized hypertonic saline hindered biofilm formation by MDR-AB and enhanced the efficacy of gentamicin and acemycin. In our experiments for the MDR-AB-producing biofilm (MDR-087 and MDR-094), the biofilm matrix seemed to contribute to drug resistance. The drug resistance associated with the biofilm matrix can be partially eliminated by aerosolized hypertonic saline. Two potential mechanisms, i.e., the alteration of stable adhesion and biofilm production, have been proposed to interrupt biofilm formation and decrease antibiotic tolerance. In addition, hypertonic saline is known to facilitate the penetration of antibiotic through the mucus layer, which may further enhance its efficacy. On the other hand, we noticed that the impacts of aerosolized hypertonic saline on susceptibility seem to be greater for MDR-ABs with higher biofilm production; one concern is that the high concentration of ions may accelerate the degradation of antibiotics. More detail studies are required to further explore the applications of antibiotic treatment with hypertonic saline aerosol. In summary, we demonstrated that using hypertonic saline aerosol to deliver antibiotics is a promising therapeutic strategy to eliminate MDR-AB infections with currently available antibiotic agents.

## Data Availability

The datasets used and/or analyzed during the current study are available from the corresponding author on reasonable request.

## References

[1] Laessig K.A. (2010). End points in hospital-acquired pneumonia and/or ventilator-associated pneumonia clinical trials: Food and drug administration perspective. Clin. Infect. Dis..

[2] Moubareck C.A., Halat D.H. (2020). Insights into *Acinetobacter baumannii*: A Review of Microbiological, Virulence, and Resistance Traits in a Threatening Nosocomial Pathogen. Antibiotics.

[3] Cillóniz C., Dominedò C., Torres A. (2019). Multidrug Resistant Gram-Negative Bacteria in Community-Acquired Pneumonia. Crit. Care.

[4] Kyriakidis I., Vasileiou E., Pana Z.D., Tragiannidis A. (2021). *Acinetobacter baumannii* Antibiotic Resistance Mechanisms. Pathogens.

[5] Kurihara M.N.L., Sales R.O., Silva K.E.D., Maciel W.G., Simionatto S. (2020). Multidrug-resistant Acinetobacter baumannii outbreaks: A global problem in healthcare settings. Rev. Soc. Bras. Med. Trop..

[6] Skariyachan S., Taskeen N., Ganta M., Krishna B.V. (2019). Recent perspectives on the virulent factors and treatment options for multidrug-resistant *Acinetobacter baumannii*. Crit. Rev. Microbiol..

[7] Wood G.C., Swanson J.M. (2017). An Update on Aerosolized Antibiotics for Treating Hospital-Acquired and Ventilator-Associated Pneumonia in Adults. Ann. Pharmacother..

[8] Flume P.A., VanDevanter D.R. (2015). Clinical applications of pulmonary delivery of antibiotics. Adv. Drug Deliv. Rev..

[9] Zhou Q.T., Leung S.S., Tang P., Parumasivam T., Loh Z.H., Chan H.K. (2015). Inhaled formulations and pulmonary drug delivery systems for respiratory infections. Adv. Drug Deliv. Rev..

[10] Wenzler E., Fraidenburg D.R., Scardina T., Danziger L.H. (2016). Inhaled Antibiotics for Gram-Negative Respiratory Infections. Clin. Microbiol. Rev..

[11] Gappa M., Steinkamp G., Tümmler B., von der Hardt H. (1988). Long-term tobramycin aerosol therapy of chronic Pseudomonas aeruginosa infection in patients with cystic fibrosis. Scand. J. Gastroenterol. Suppl..

[12] Chen Y.M., Fang W.F., Kao H.C., Chen H.-C., Tsai Y.-C., Shen L.-S., Li C.-L., Chang H.-C., Huang K.-T., Lin M.-C. (2014). Influencing factors of successful eradication of multidrug-resistant Acinetobacter baumannii in the respiratory tract with aerosolized colistin. Biomed. J..

[13] Lu Q., Luo R., Bodin L., Yang J., Zahr N., Aubry A., Golmard J.-L., Rouby J.-J., Nebulized Antibiotics Study Group (2012). Efficacy of high-dose nebulized colistin in ventilator-associated pneumonia caused by multidrug-resistant Pseudomonas aeruginosa and Acinetobacter baumannii. Anesthesiology.

[14] Badave G.K., Kulkarni D. (2015). Biofilm Producing Multidrug Resistant Acinetobacter baumannii: An Emerging Challenge. J. Clin. Diagn. Res..

[15] Davies D. (2003). Understanding biofilm resistance to antibacterial agents. Nat. Rev. Drug Discov..

[16] Flemming H.C., Wingender J., Szewzyk U., Steinberg P., Rice S.A., Kjelleberg S. (2016). Biofilms: An emergent form of bacterial life. Nat. Rev. Microbiol..

[17] Lee H.W., Koh Y.M., Kim J., Lee J.-C., Lee Y.-C., Seol S.-Y., Cho D.-T. (2008). Capacity of multidrug-resistant clinical isolates of Acinetobacter baumannii to form biofilm and adhere to epithelial cell surfaces. Clin. Microbiol. Infect..

[18] Meyer B. (2003). Approaches to Prevention, Removal and Killing of Biofilms.

[19] Zapotoczna M., O’Neill E., O’Gara J.P. (2016). Untangling the Diverse and Redundant Mechanisms of Staphylococcus aureus Biofilm Formation. PLoS Pathog..

[20] Chen C.S., Shiu R.F., Hsieh Y.Y., Xu C., Vazquez C.I., Cui Y., Hsu I.C., Quigg A., Santschi P.H., Chin W.-C. (2021). Stickiness of extracellular polymeric substances on different surfaces via magnetic tweezers. Sci. Total Environ..

[21] Guttenplan S.B., Kearns D.B. (2013). Regulation of flagellar motility during biofilm formation. FEMS Microbiol Rev..

[22] Tsuneda S., Aikawa H., Hayashi H., Yuasa A., Hirata A. (2003). Extracellular polymeric substances responsible for bacterial adhesion onto solid surface. FEMS Microbiol. Lett..

[23] Guo Y.S., Furrer J.M., Kadilak A.L., Hinestroza H.F., Gage D., Cho Y.K., Shor L.M. (2018). Bacterial Extracellular Polymeric Substances Amplify Water Content Variability at the Pore Scale. Front. Environ. Sci..

[24] Lin H.L., Chiu L.C., Wan G.H., Huang C.-C., Lee Z.-T., Lin Y.-T., Wu S.-R., Chen C.-S. (2020). Hypertonic saline enhances the efficacy of aerosolized gentamicin against Pseudomonas aeruginosa. Sci. Rep..

[25] Harimawan A., Rajasekar A., Ting Y.P. (2011). Bacteria attachment to surfaces–AFM force spectroscopy and physicochemical analyses. J. Colloid Interface Sci..

[26] Klemmer A., Krämer I., Kamin W. (2013). Physicochemical compatibility of nebulizable drug admixtures containing budesonide and colistimethate or hypertonic saline. Int. J. Pharm. Compd..

[27] Carro L.M., Martínez-García M.A. (2020). Use of Hyaluronic Acid (HA) in Chronic Airway Diseases. Cells.

[28] Reeves E.P., Williamson M., O’Neill S.J., Greally P., McElvaney N.G. (2011). Nebulized hypertonic saline decreases IL-8 in sputum of patients with cystic fibrosis. Am. J. Respir. Crit. Care Med..

